# Microservices in Web Objects Enabled IoT Environment for Enhancing Reusability

**DOI:** 10.3390/s18020352

**Published:** 2018-01-26

**Authors:** Muhammad Aslam Jarwar, Muhammad Golam Kibria, Sajjad Ali, Ilyoung Chong

**Affiliations:** Department of Information and Communications Engineering, Hankuk University of Foreign Studies, Seoul 02450, Korea; aslam.jarwar@hufs.ac.kr (M.A.J.); kibria@hufs.ac.kr (M.G.K.); sajjad@hufs.ac.kr (S.A.)

**Keywords:** Web of Objects (WoO), microservices for IoT, objects reusability, Internet of Things (IoT)

## Abstract

In the ubiquitous Internet of Things (IoT) environment, reusing objects instead of creating new one has become important in academics and industries. The situation becomes complex due to the availability of a huge number of connected IoT objects, and each individual service creates a new object instead of reusing the existing one to fulfill a requirement. A well-standard mechanism not only improves the reusability of objects but also improves service modularity and extensibility, and reduces cost. Web Objects enabled IoT environment applies the principle of reusability of objects in multiple IoT application domains through central objects repository and microservices. To reuse objects with microservices and to maintain a relationship with them, this study presents an architecture of Web of Objects platform. In the case of a similar request for an object, the already instantiated object that exists in the same or from other domain can be reused. Reuse of objects through microservices avoids duplications, and reduces time to search and instantiate them from their registries. Further, this article presents an algorithm for microservices and related objects discovery that considers the reusability of objects through the central objects repository. To support the reusability of objects, the necessary algorithm for objects matching is also presented. To realize the reusability of objects in Web Objects enabled IoT environment, a prototype has been designed and implemented based on a use case scenario. Finally, the results of the prototype have been analyzed and discussed to validate the proposed approach.

## 1. Introduction

In the Ubiquitous Internet of Things (IoT) environment, millions of objects are connected to the web, for intelligent and user-preferred IoT service provisioning. It is predicted that numbers of objects will exponentially increase to billions in the coming future, and may create problems of objects duplication, communication, deployment, maintenance, and data abundance. The reusability of objects will minimize the cost of recreation, storage, and maintenance. An efficient and effective mechanism of reusing of already available objects and their data may overcome the problems, and improve the smartness of objects for more intelligent IoT services. The reusability of objects across the different platforms has been discussed in horizontally layered architecture platforms such as oneM2M [1]. The horizontally layered platforms do not address the specific issues in a specific domain but they support necessary tools to resolve problems of cross-cutting services across the platforms. However, vertically layered architectures have been commonly used to resolve domain specific problems [2,3]. Due to the special features of vertically layered architectures in resolving the domain specific issues, this article is proposing the reusability of objects in vertically layered architecture by using microservices. Microservices in vertically layered architecture improves service modularity, extensibility, availability, and scalability of IoT services by reusing the existing objects. To resolve these issues and to use the advantages of microservices in IoT, this article proposes microservices in Web of Objects (WoO) enabled IoT environment for reusability of objects. WoO is an efficient IoT platform for domain specific solutions as well as cross-domain reusability issues. The vision of WoO is to provide simple but an efficient platform for the intelligent IoT services [4]. In WoO platform, Virtual Object (VO) and Composite Virtual Object (CVO) harmonize the real world objects by using semantic web technologies for relating, understanding, and exchanging of data among multiple IoT application domains. Nowadays, smart homes, smart agriculture, smart grids, smart spaces, cyber-physical systems for autonomous vehicles, smart city, smart manufacturing, and smart healthcare are the most popular IoT application domains [5]. To provide better and intelligent IoT services, it is necessary to foster the reusability of objects in various WoO based IoT application domains. When these large numbers of objects are reused in IoT environment, it raises many important questions. How will these objects be reused; how will these objects support plug and play functionality; how will these objects be related to other domain objects; and how will these objects be shared and exchanged must be answered so that they provide functionalities needed by the IoT applications [6]. The research and techniques that facilitate the reusability of objects from various application domains to communicate, create and share information will change the physical world into a highway of information and knowledge. This highway of information and knowledge in IoT is not only connecting objects from the physical world to the web for communication and linking channels but this linking and communicating phenomena will also enhance the smart capability of objects [7]. Microservices based architecture supports developing a service as a group of small services [8], and this pattern supports the reusability of microservices in multiple IoT services. Microservices have the enormous support of structuring the IoT environment over the web, for creating, connecting and reusing the information highway as a collection of loosely coupled services in terms of microservices. The microservices based architecture further enables the rapid development and deployment of large and complex applications, and supports uninterruptable delivery of services [9]. However, domain knowledge plays an important role to use microservices pattern in the architecture of large and complex systems. 

Currently, 13 billion objects are considered as connected objects and it is expected that the numbers of connected objects will reach 30 billion in 2020 [10]. This increase in a huge number of objects for current and future IoT applications creates many challenges and opportunities for the academic, and industries working in the IoT area. In the broader concept and vision of IoT in the future, every connected object will be reused in multiple IoT application domains for enhancing the smartness of IoT services. For example, in the fourth industrial revolution, the manufacturing industries cooperate with cyber physical systems and other industries around the world to produce cost effective things by providing data exchange and real time access to the product and production information [11]. For reusing and fostering the cooperation among objects in multiple IoT application domains, we need to create semantically interoperable application programming interfaces (APIs). These semantic interoperable APIs should be developed with the best fit technologies to support the reusability of objects, rapid development, deployment, recovery, and resiliency.

Among these challenges, the following are the main and problem statements of this article:

- How microservices, CVOs, and VOs are searched, shared, and reused among different application domains in WoO enabled IoT environment for IoT service provisioning;
- How to consider the communication and complexities of semantic cooperation between microservices, CVOs, and VOs in their own application domain and as well as in other domains, for enhancing the reusability of objects and extensibility; and
- How to support the functionalities of every connected object and IoT service provisioning rapidly implemented in best fit and lightweight technologies, deployed independently, with resiliency and recoverability in minimum time.

WoO articles [12,13,14,15] have not provided the mechanism of communication, reusing and understanding of information among various WoO based IoT application domains. In Reference [16], authors have presented the service modularity by reusing VOs in WoO platform but the core of service modularity that how the service will be exposed and reused in multiple application domains has not been addressed. This article presents the approach of discovering, exposing, matching, mapping, and reusing of objects in terms of CVOs through microservices, which can be accessed and shared among multiple IoT application domains enabled with WoO platform. 

The following are our main contributions that support the above-mentioned challenges:

- Using and discovering microservices, CVOs, and VOs in WoO enabled IoT environment;
- A central objects repository microservice and objects mapping and aligning microservices; and
- A WoO design and development based on microservices pattern. As microservices support the proposed design and technological requirements with objects reusability, plug & play, best fit, and lightweight features.

The paper is organized as follows. Section 2 presents background and related work. Section 3 discusses web of objects architecture for IoT service provisioning. Section 4 elaborates a functional model of microservices in WoO platform. Section 5 presents enhancing the reusability of objects in WoO based IoT environment. Section 6 discusses use case and prototype. Section 7 discusses experiments and results. Finally, Section 8 concludes the article.

## 2. Background and Related Work

Since the early days of IoT, most of the IoT platforms are using monolithic approach for system designing and implementation, and also followed the same approach for the provisioning of IoT services. In monolithic approach, services are developed and updated separately and recurrently for different users, which increase the overall cost of service development, deployment, and maintenance. This cost can be minimized by distributing the functionality of a large IoT service into a group of several small services. These multiple small services can be combined and reused in a big IoT service; with more features and more customizability. The term of microservice was coined in 2011 and become a hot topic in 2014 [17,18]. Microservices pattern is the extension of service oriented architecture (SOA). Microservice approach is the best way to scale SOA based systems [19]. In IoT environment, SOA and microservices style services are used to expose the real world objects on the web and providing a mechanism of creation of services from the group of small services. Microservices have been widely discussed in cloud computing environment service provisioning. Few studies [20,21,22] have discussed the significance and microservices architecture for building IoT applications and platforms. 

In Reference [21], authors discussed interoperable IoT platform by using the concept of microservices. The aim of this article is the provision of communication among the heterogeneous IoT devices. Authors modularized their proposed framework based on layering architecture. The bottom layer used for the load balancing of IoT nodes and the microservices at middle layer performed the tasks of data processing, data aggregation, and data transformation by using microservices pipeline. For building smart cities, the microservices based IoT platform proposed in Reference [22]. In this IoT platform, authors distributed the functionality of large system into many microservices. These microservices performed the tasks of data collection, data aggregation, and data analytics on live streaming data. Sun et al., (2017) [23], compared the monolithic approach and microservices approach of system designs for building IoT platforms. The comparison showed that microservices approach is more useful than the monolithic. In their system design, authors proposed microservice for geospatial data correlations to render location data on the map. Authors have also created microservice for user management functions.

In a wireless sensor network, tiny IoT objects distributed across the large sensing filed. Most of the energy of these tiny sensors consumed in transmitting data from the node to a central point. Microservices implementation in these tiny objects can increase the life time by aggregating and processing the data at the node level and then it sends the aggregated information to the central point [24]. In our proposed approach, lightweight microservices fetch objects from other application domains and reuse them similar to their own domains. For IoT system functionalities and services development, the microservices pattern is useful. Microservices pattern supports service modularity, single task executability and functional independency [25]. 

Microservices pattern supports object virtualization [26]. In the microservices pattern, virtualization is supported by isolated execution environment for every microservice. Microservice interacts with other microservice with lightweight payloads and HTTP-REST protocols. The communication mechanism among the microservices was achieved by explicit interfaces and protocols. “The entire idea of microservices becomes plug and play” [27]. The composability feature of microservices supports to create IoT applications from a group of microservices [25], and this feature increases the reusability of microservices. Composability facilitates the fine-grained arrangement of microservices through already available instances of microservices. Once composed microservices with RDF and JSON payload can be reused in various application domains for service provisioning in IoT environment.

The microservices approaches discussed in the above literature work does not provide semantic interoperability at the service level to meet the service provisioning request from various IoT application domains. As the semantic interoperability is a key requirement of future intelligent IoT services. Therefore, our proposed approach has been using microservices for object reusing and object sharing among various application domains at the service level. Microservices patterns are also used in WoO architecture design and development of functional components by incorporating semantic web technologies. 

In our study for enhancing the reusability of objects with microservices, we need semantic interoperability for aligning and mapping of objects among different application domains in WoO based IoT environment through microservices and semantic technologies. “Semantic interoperability means enabling different agents, services, and applications to exchange information, data, and knowledge in a meaningful way, on and off the Web” [28]. In IoT environment, semantic interoperability cannot be achieved without setting the foundations of interoperability, i.e., technical interoperability and syntactic interoperability. Technical interoperability is achieved through setting a standardized organization wise mechanism for each object and services in IoT applications. Solutions for technical interoperability were discussed in Global Sensor Networks (GSN) Framework, SENSEWEB Platform, and FOSSTRAK tools [29]. The requirement of syntactic interoperability can be achieved by clearly and agreeably defined data and interfaces between client and server. When the object data are exchanged and reused among various application domains in WoO enabled IoT environment, it should be in well-defined formats, e.g., JSON, XML, and RDF. The reusing of devices and services has been widely discussed in horizontal IoT architectures. In horizontal IoT standards, oneM2M is the most popular. The main goal of the oneM2M standard is to define independent accessibility interfaces for M2M services across the IoT platforms [30]. The oneM2M standard supports the IoT architecture with three layers: applications, services, and networks. In Reference [31], authors discussed the ontology over the oneM2M base ontology for achieving the semantic data interoperability at services level in the heterogeneous IoT environment. To break the boundaries, Lysis platform used the iCore vertical architecture and defines the social relationships among the virtual objects for fostering the horizontal IoT [32]. The Lysis also considered the reusability at different layers in the architecture; in terms of object templates, third party services, and codes. The reusability feature in terms of software implemented in Xively platform, so the community can share the firmware for common devices but it did not provide the reusability at service level for reusing the services and the data [33].

In Reference [34], authors have used ontology based semantic queries to detect user indoor activities within the boundaries of a university campus. University activity ontology (UAO) was used in their ontology based query model for sharing, exchanging, and reasoning to support semantic relations among the classes. In Reference [35], authors discussed a semantic data-driven platform for the annotation and reasoning of IoT data. This semantic data-driven approach used ontologies to present contextual information and annotations of raw sensor data. The service was used as semantic web gateway for enabling interoperability among systems [36]. The semantic web gateway translates messages between services by using multi-protocol proxy architecture. A research article [37], proposed semantic annotations of data received from smart objects. The semantic annotation of smart object’s data was performed to integrate smart objects with IoT applications through the web. 

## 3. Web of Objects Architecture for IoT Service Provisioning

**Figure 1:**
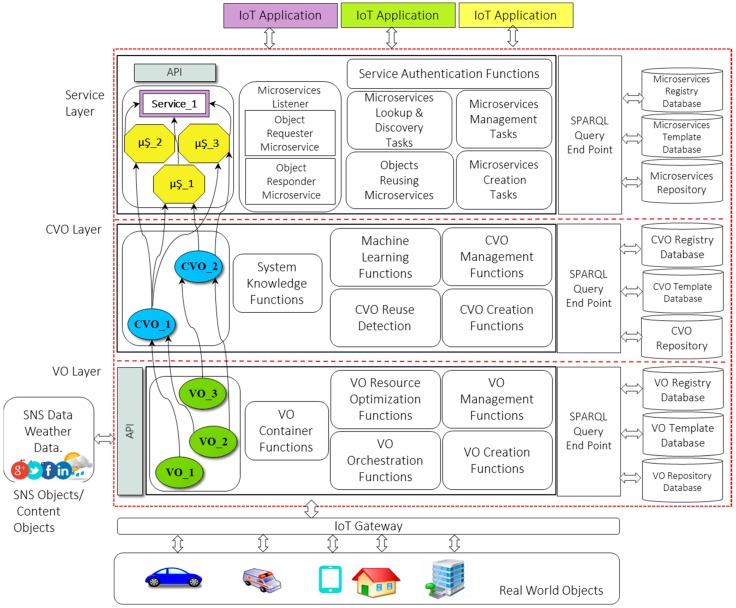
Microservices based IoT service provisioning in Web of Objects architecture.

WoO is the IoT platform which provides a way to virtualize the real world objects (RWOs) and content objects through VOs. The semantic rules are applied over the mashup of VOs and CVOs for the provisioning of IoT services from the group of microservices. The mashup of VOs is called a CVO, and the conglomeration of CVOs is called a microservice. The standalone microservice can be reused in two different IoT services and a user can also choose few microservices from the group of microservices for the creation of new IoT services. The VOs are connected, controlled and incorporated with the RWOs, social network service objects or any other content objects. VO supports development, deployment, and functional operations for IoT services on the World Wide Web. WoO supports the representation of RWOs and content objects as “web objects” on the web through the VO. It provides the core functionalities of creating, deploying, and maintaining of IoT services from the group of microservices. The microservices based Web of Objects layered architecture is shown in Figure 1. WoO architecture has three layers: VO layer, CVO layer, and Service layer.

The VO layer provides the API to connect RWOs and content objects to WoO platform. VO layer also provides API for sending and receiving of data from third party services. These third party services can be social network services (SNS), or weather services (WS). To connect third party services with WoO platform has great importance, as the data from these services can be reused for the customization and user preferred IoT services [38].

The virtual object is the key component in IoT platforms for digital representation of RWOs, service discovery and service provisioning [39]. To virtualize RWOs with VO was discussed in iCore [40,41]. In iCore, the VO can represent ICT (Information and Communication Technologies) object and non-ICT object and the VO in WoO can be sensor, device, task, process, and information. The VO in WoO contains semantic ontology for semantical representation of data generated by RWOs. By using VO and CVO ontology, actionable knowledge is created for triggering the actions on RWOs. The VO provides a semantic data model, and semantic data annotation schemes to harmonize RWOs and represent content objects in WoO platform.

The VO registry database holds the current status of VOs and performs the task of record keeping. The record keeping includes metadata of VO type, VO creation date, and duration for which the particular VO was used. A VO template is created so the other similar types of RWOs can easily be represented. The VO template is a basic description and properties to represent the RWOs and content objects. The VO template is created and stored as OWL (Web Ontology Language) file. In WoO platform, the VO template file includes the RDF and XML tags for the VO profile, VO temporal features, and RWOs attributes. The VO template database holds the default or initial VO templates. The initial VO template is used in the situation when the new object requests to connect to the WoO platform. Upon connecting a new object, the default template updated as per the specification of that object. The default or initial template is created from the VO information model [16]. The VO management module includes the functions for changing and updating of VOs, VO access rights, and VO lookups. The VO management functions also include the capability to resolve the triggering action conflicts; in this case, the VO represents the actuator. 

The CVO applies semantic rules on the selected list of VOs, for the execution of service features with microservices. In WoO platform, RWO and other content object’s data are semantically annotated through VOs to achieve actionable knowledge. This actionable knowledge is used to monitor real world situations; for example, receiving current temperature, current humidity, current weather update, and detecting the occupancy of persons in the meeting room to automatically adjust room temperature. In the example, for the questions of when, why, how and at what level room temperature could be adjusted, the CVO at CVO layer decides the answers. The CVO in this example can be “MEETING_ROOM_HAVC_MONITORING”.

To support service modularity and scalability, the CVO layer functions and database are separated into functional groups by following microservices patterns. The CVO layer includes functions to support the operations of CVO layer. These functions include CVO management functions, CVO creation functions, machine learning functions, CVO reuse detection functions, and CVO system knowledge functions. The CVO management functions contain CVO update, CVO authorize, CVO discover, and CVO search functions. Machine learning functions are required to extract and inference the knowledge from semantically annotated data. The CVO reuse functions have great importance in WoO platform. The CVO reuse detection function triggered when the graph of CVOs (RDF/OWL payload) received from service layer for the execution of service features using microservices. This function checks for already available CVO instances which are executed, which executions can be reused to fulfill service requests, or which need to create a new instance. The system knowledge functions collect facts about the CVOs which are used in the execution of service features. These functions are used by the domain expert or system developers to create/update new CVO templates and also used by the internal system components to execute service features through microservices. The explanation of service layer (Figure 1) will be carried out in next section along with microservices algorithm and microservices information model.

## 4. Functional Model of Microservices in WoO Platform

**Figure 2:**
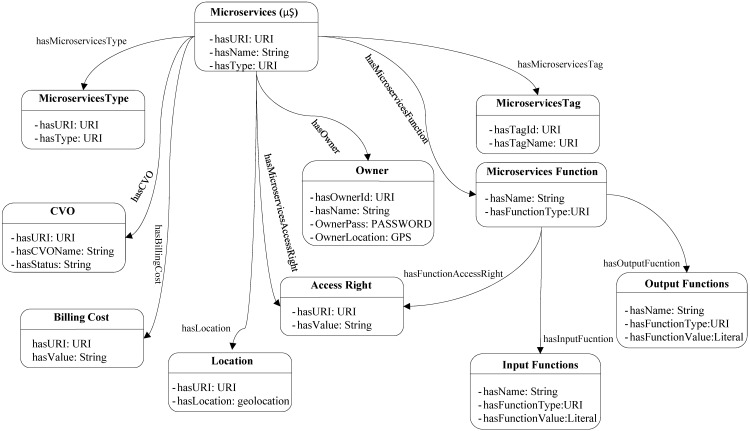
Information model for microservices.

The service layer in WoO platform provides the northbound interfacing API for the IoT applications. For enhancing the reusability of objects microservices, listener and object reusing microservices have been introduced at the service layer (Figure 1). Microservice listener performs the tasks of requesting a missing object from the central object repository as well as responding to the requested objects from other WoO enabled application domains. The phenomena of requesting and responding to objects are performed with the coordination of central object repository microservice. The request of IoT service can be fulfilled with a single microservice or with a group of microservices. The service layer contains databases and service level functional components to foster IoT services modularity, scalability, and plug and play functionality with microservices. The microservices registry database holds the status of current microservices. This status can be microservices execution time, expected finish time, and how many times a microservice is used in which service request. The microservices template database contains initial microservices templates from which other microservices will be created. The microservices repository database stores the created microservices templates. The created microservices template includes microservices metadata and the list of CVOs to execute service features with microservices. The microservice template is created based on the microservice information model. The information model for microservices is shown in Figure 2.

Microservices information model includes several functional, non-functional and descriptive properties, such as input and output preconditions. Microservices information model provides well-defined and standardized metadata; that offer all necessary functionalities for interacting with the CVO layer. The microservices information model also includes billing cost for the advertising, accessing and using of microservices. Access rights restrict accessing both microservices and its functionalities because a user might be allowed to access a microservice, but all the functionalities might not be allowed to access and use it. Input and output functions describe the incoming and outgoing properties and functionalities of microservices.

Microservices supports service layer functions and tasks. These tasks and functions contain microservices management tasks, microservices creation tasks, microservices lookup and discovery tasks, and service authorization functions. The other components include objects reusing microservices, and microservices listener, for requesting and responding of objects from/to other application domains through central object repository microservice. When a service request arrives at service layer from the IoT applications, the service authentication function checks the service credentials. After service request authentication, microservices lookup and discovery functions are used to search required microservices from the microservices registry for the execution of service request. The object reusing microservices reuses the already instantiated object from the local domain registry to fulfill the service request and also checks local domain registry from time to time for the newly created or updated object. The information of newly created or updated object disseminated to the central objects repository in the cloud through the central objects repository microservice and object reusing microservices.

**Figure 3:**
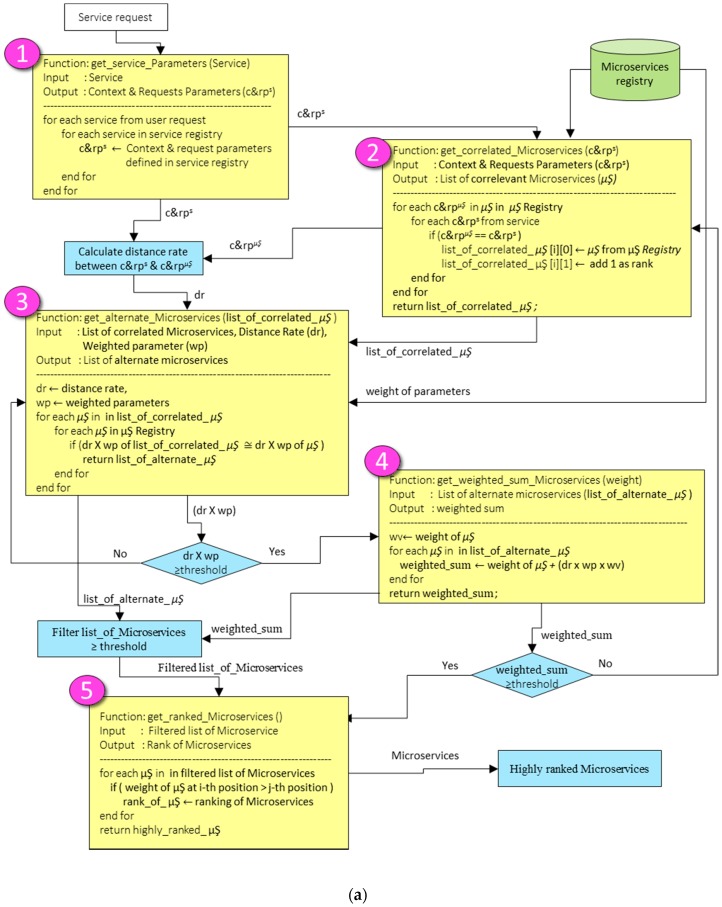

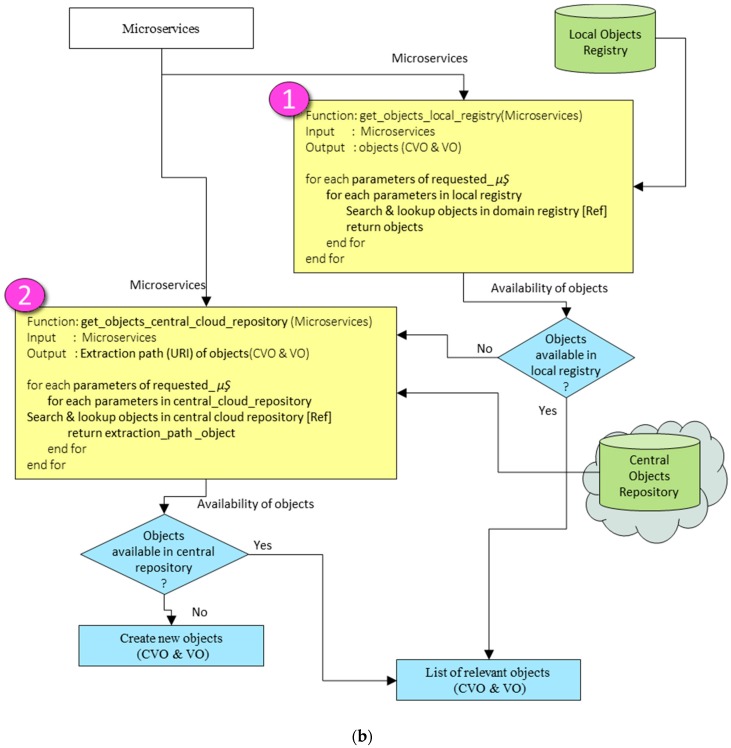
(**a**) Microservices discovery and lookup algorithm; and (**b**) microservices relevant objects discovery and lookup algorithm.

Microservices and related objects discovery algorithm are shown in Figure 3a,b. This flow chart based algorithm searches microservices and required objects (i.e., VO, CVO) from local domain registry. If the required objects are not found in local domain registry, then microservices create a request to the central objects repository. The process of microservices and related objects discovery is explained as follows:

- When the service is requested from the IoT application, the *get_service_parameters* function takes input as a service request.
- The function *get_service_parameters* searches the service in service registry based on service input parameters and it returns the matched Context and Requests Parameters (c&rp^s^) from the service registry.
- Then, the correlated microservices are discovered from the microservices registry. The discovery process is based on the Context and Requests Parameters (c&rp^s^). At this step, the *get_correlated_microservices* function is used. The correlated microservices are those that are matched based on the initial service request.
- After the discovery of correlated microservices (c&rp^µŞ^) and Context and Requests Parameters (c&rp^s^), the distance rate between c&rp^s^ and c&rp^µŞ^ is calculated.
- The function *get_alternate_microservices* takes three inputs from the microservices registry: distance rate (*dr*), list of correlated microservices (*list_of_correlated_µŞ*) and weight parameters (*wp*). This function returns a list of alternate microservices. The list of alternate microservices is the group of microservices which are more filtered microservices and are matched with the service request.
- The product result of distance rate (*dr*) and weight parameters (*wp*) is compared with a threshold value. If the result of the product is greater than or equal to a threshold value, then the group of alternate microservices is selected; otherwise, the list of alternate microservices will be obtained by using the *get_alternate_microservices* function.
- From the group of selected alternate microservices, the weighted sum of all microservices is calculated with the *get_weighted_sum_microservices* function.
- The *get_ranked_microservices* function ranks all the microservices from the filtered list and returns highly ranked microservice from the group of filtered microservices.
- Now, the filtered microservice checks its objects (i.e., VO, CVOs) to execute the service features.
- In Figure 3b, the *get_objects_local_registry* function checks the required objects in the local object registry. If the required objects are not found in the local object registry, then the *get_object_central_cloud_repository* function will get required objects from the central objects repository by using central object repository microservice.

## 5. Enhancing Objects Reusability in WoO Based IoT Environment

**Figure 4:**
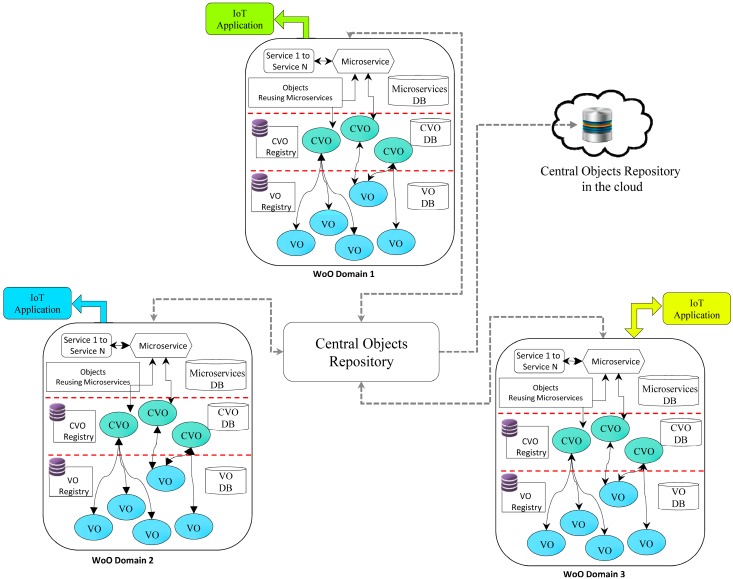
Objects reusability and extensibility in WoO enabled IoT environment.

In the previous sections, we have discussed microservices functional model in WoO platform for IoT service provisioning and using a group of microservices to execute IoT service request. Now, problems of reusing and extending required or customized objects of other application domains are considered. The phenomena of sharing, understanding and reusing of objects in various WoO enabled application domains through microservices are shown in Figure 4. The mechanism presented in Figure 4 fosters the reusability of objects, extensibility, and creation of complex IoT applications by combining the information and knowledge from simple small IoT application domains.

Three WoO based IoT application domains are shown in Figure 4. Each domain contains VO, CVO, and service layer. The service layer contains microservices and microservices related functions. Each application domain implementation has two northbound interfaces. The first is known as service API and the second is called a microservices listener. The service API is used for the service requests from the IoT applications to the WoO platform. For example, in the smart home application domain, the service request from IoT application can be a controlling and monitoring of room heating, cooling, and ventilation.

The microservices listener contains Object Requester Microservice (OReqµŞ) and Object Responder Microservice (OResµŞ). The OReqµŞ and OResµŞ are used to handle the communication mechanism for requesting and responding of objects from central objects repository in WoO enabled application domains. The objects reusing microservices functional block contains the microservices for processing and mediating of objects. Thus, these objects can be reused in the requested application domain.

For clearly understanding of the following explanation, we consider the domain which makes a request for the object as domain_requester, and the domain whose object will be served is considered as domain_requestee.

When the IoT application sends a service request to its own domain and domain microservices do not meet the requirements of objects to serve a request, or the domain wants to reuse the customized objects of other domain through the central objects repository in the cloud, then the object requester microservice (OReqµŞ) will make a request to the central objects repository microservice. Central objects repository microservice lookups its repository, and will serve the best object to the requester. As the central objects repository microservice is using the weight factor for each object stored in the cloud. The domain_requester objects reusing microservices will receive the object, and process and mediate that object according to their own domain ontologies and system configuration. The detail process of objects reusing microservices is explained in the following section.

### Objects Matching and Alignment

The received response from the central object repository is processed and mediated by the domain_requester microservices so the received object can be reused in the domain_requester. The object reusing microservices at domain_requester can semantically understand the received object and fulfill the deficiency of objects for the execution of service features. The object matching microservices receives payload as a response from the central objects repository microservice. This payload is then sent to the filtering microservices for further processing. The objects filtering microservices filters the payload into entities. These entities contain domain_requestee concepts used in the required object and object metadata. By using concepts of the received object from the domain_requestee and domain_requester concepts, microservice (µŞ_a) and microservice (µŞ_b) compare both concepts. The matching and comparison of concepts are performed based on domain ontology properties (i.e., object properties, data properties, and equivalent properties). Microservices µŞ_c and µŞ_d check for ambiguous terms, synonym, hypernyms, replacement, and term equivalency between two object’s concepts of different application domains (domain_requester and domain_requestee) from the central vocabulary and WordNet dictionary, respectively. To identify a matched object from two different application domain ontologies, a matching decision has been carried out on the score of several matching concepts. The matching cumulative score between the two objects concepts is expressed in Equation (1). In the equation, the requested CVO ($CVO\_Concepts^{req}$) is matched and aligned with the responded CVO ($CVO\_Concepts^{res}$) based on CVO ontology concepts, properties and relationships. The final matching decision will take place based on a threshold value. The process taking place in these microservices is explained in Algorithm 1.

(1) $$\begin{array}{l} match\left(CVO\_Concepts^{req},CVO\_Concepts^{resp}\right)\\ =\{\begin{array}{ll} 1, & \frac{1}{n}\overset{n}{\underset{k=1}{\sum }}\left|matchfunction\left(CVO\_Concepts^{req},CVO\_Concepts^{resp}\right)\right|\;\geq {\mathrm{threshold}}_{\mathrm{match}}\\ 0, & otherwise \end{array} \end{array}$$

**Algorithm 1.** Algorithm for matching and aligning**Input domain_requester_O**_I_**,**
$\;CVO^{resp}$**Output domain_reqester_CVO** ($CVO^{req}$) **and CVO minimum matched Value**1:**function** Get_domain_requester_CVO(O_I_, $CVO^{resp}$)2:  ● $CVO^{resp}\to$ responded CVO from the repository3:  ● O_I_
→
*domain_requester* ontology4:  ● F_I_
→ Features of Ontology O_I_5:  ● F_J_
→ Features from O_J_6:  ● U_FO_
→ Uniform concepts for the required CVO7: F_I_ ← getFeatures(O_I_)8: F_J_ ← getFeatures(CVO^resp^)9: **for**
∀ F_J_ in F_I_
**do**
10:  **if** match(F_J_, F_I_) ≥ 1 //match concepts domain ontology11:  **then** M_F1_ ← getMatchedFeature (F_J_, F_I_)12:  **else if** Match(F_J_, F_I_) = 013:  **then**
¬M_F1_ ← getNotMatchedFeatures(F_J_,  F_I_)14:  **end if**15:   **if** (check and Replace ¬M_F1_ in central vocabulary) ≥ 116:   **then** M_F2_ ← getMatchedFeatures(¬M_F1_)17:   **else if** Match(¬M_F1_) = 018:   **then**
¬M_F2_ ← getNotMatchedFeatures(¬M_F1_)19:   **end if**20:    **if** (check and Replace ¬M_F2_ in WordNet Dictionary) ≥ 121:    **then** M_F3_ ← getMatchedFeatures(¬M_F2_)22:    **end if**23:  Score ← $\frac{1}{3}$
$〚Sum\{count\left(M_{F1}\right),count\left(M_{F2}\right),count\left(M_{F3}\right)\}〛$24:     **if** Score ≥ threshold25:     **then**
$CVO^{req}$ ← merge($M_{F1}$, $M_{F2}$, $M_{F3}$)26:     **end if**27: **end for**28:      // For requested and responded CVOs $CVO^{req}$, $CVO^{resp}$29:       **If**$CVO^{req}$, $CVO^{resp}$ are similar30:       **then** sim_distance($CVO^{req}$, $CVO^{resp}$) = 131:        **and return**
$CVO^{req}$_,_ 132:       **else** check for the %age of matching between $CVO^{req}$, $CVO^{resp}$33:   CVO_min_matched_value_ = sim_distance($CVO^{req}$, $CVO^{resp}$) = $\sum^{\text{​}}dist\_min\left(CVO^{req},CVO^{resp}\right)$34:  **return**
$CVO^{req}$, $CVO_{min\_match\_value}$35:**end function**

Algorithm 1 explains the process taking place to match and align the objects for enhancing the reusability of objects with microservices. The algorithm takes two inputs, i.e., domain_requester ontology (O_I_) and responded CVO ($CVO^{resp}$). The responded CVO ($CVO^{resp}$) contains the ontology concepts related to the original application domain. Initially, the features of domain_requester ontology (e.g., concepts, properties and relationship) and responded CVO ($CVO^{resp}$) are extracted. Then, the features of CVO ($CVO^{req}$) and CVO responded ($CVO^{resp}$) are matched. The matched and non-matched features are extracted in this step. The non-matched features are processed further for matching with the help of ontology concepts, wordNet dictionary, and central vocabulary. After matching, the matched features are counted for each matching steps and the average matched CVO features are converted to score. Based on the threshold score, all matched concepts, properties, and relationships are aligned and merged for making it a single requested CVO ($CVO^{req}$). To check the minimum match value of requested and finally harmonized CVO from both concepts, the similarity distance has been calculated with two well-defined similarity distance techniques: Levenshtein edit distance (LD) [42,43], and Leacock-Chodorow matcher (LCM) [44,45]. Finally, the requested CVO ($CVO^{req}$) and the minimum matched value have been returned to the microservices. Then, the domain_requester microservices will use the received object to fulfill the service request. 

## 6. Use Case and Prototype

Microservices in WoO architecture supports the enhancement of object reusability and extensibility in WoO enabled IoT environment. WoO provides IoT services from the group of microservices and enables the ubiquitous IoT environment for user smart space. A smart space based IoT applications offer intelligent IoT services based on user location, context, and activity in a seamless manner. In these smart space applications, objects are shared, and reused in the case they are not available at the existing domain or the services need more customized objects. To realize the development of objects’ reusability and discovery for fulfilling the requirement of service request, a use case has been designed and a prototype has been implemented.

### 6.1. Use Case Scenario

The use case scenario involves a user living in a home equipped with the smart features based on WoO platform. This system provides smart home features (i.e., heating, lighting, air-conditioning, security, fire detection and management) and user health monitoring services. The user needs to visit other cities frequently for her business trips. During her business trips, she stays in the hotel, and she wants the same comfort and satisfaction that she has at home. As a laywoman, she does not know how to create a service from a group of microservices and share the objects between her home and hotel room. The hotel also provides the room heating and air-conditioning services based on WoO platform, but it has different objects. From this use case, two IoT application domains (smart home and smart hotel) have been identified. 

### 6.2. Proof of Concept

The settings of user preferred and customized services in living room environment depend on user context, user health, user activity (e.g., sleeping, reading, and listening), and weather condition. Smart home domain features include home environment monitoring, safety monitoring, and emergency situation monitoring. Smart hotel domain features include guest safety and emergency situation; it also provides room environment monitoring with general rules and logic. The rules and logic of services provided by the smart hotel application domain are applicable to all guests. Therefore, the smart hotel domain microservices need to request users preferred and customized objects from smart home domain to execute service features, as requested by the user. As discussed earlier, the user wants her preferred and customized comfort level at hotel room environment, and health monitoring services that she has at home.

**Figure 5:**
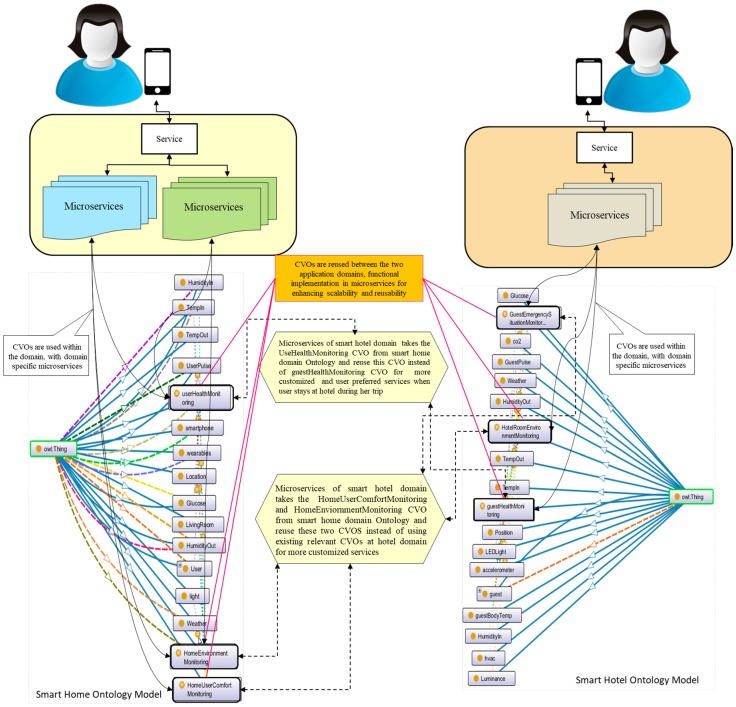
Microservices with relevant CVOs and VOs in ontology for smart home and smart hotel as a proof of concept of reusing the CVOs for customized and user preferred services.

When the user selects and activates services (i.e., health monitoring service, room environment monitoring service, safety monitoring service, and emergency situation handling service) by using her smartphone, microservices of smart hotel domain will make a request to the smart home microservices for the objects. In this scenario, we assume that the user already grants the permission for reusing of objects. These objects include rule, logic, and metadata to execute service features according to the user preferences and customization. After retrieving the objects from the smart home domain, microservices at smart hotel domain will use these objects with their own VOs, and CVOs. The VOs represent relevant sensors and actuators in smart hotel domain. These sensors include indoor and outdoor temperature sensors, CO_2_ sensor, humidity sensor, luminous sensor, light sensor, position sensor, accelerometer sensor, plus sensor, glucose sensor, and body temperature sensor. These actuators include HAVC, LED, alarm, fan, digital signage, etc. Figure 5 shows microservices model with relevant CVOs and VOs in the ontology for smart home and smart hotel application domains as a proof of concepts.

In Figure 5, the ontology model of smart home and smart hotel application domains have been shown with the microservices. Smart home ontology model and smart hotel ontology model contains VOs, CVOs, and microservices separately. Smart home ontology model contains three CVOs (i.e., UserHelathMonitoring, HomeEnviornmentMonitoring, and HomeUserComfortMonitoring) and two microservices with many instances for the execution of CVO functionalities and load balancing of services. The smart hotel ontology model also contains three CVOs (i.e., GuestEmergencySituationMonitoring, HotelRoomEnviornmentMonitoring, and GuestHealthMonitoring), a microservice with many instances and many VOs. These three CVOs from the smart home ontology model are used with the application domain microservices in the respective domain, and reused in smart hotel domain microservices for more customized and user preferred IoT services, when the same user stays in a hotel during her business trip. The proof of concept shows that the CVOs are reused and extended to other application domains for better services without creating new CVOs for that domain.

### 6.3. Prototype Details

**Figure 6:**
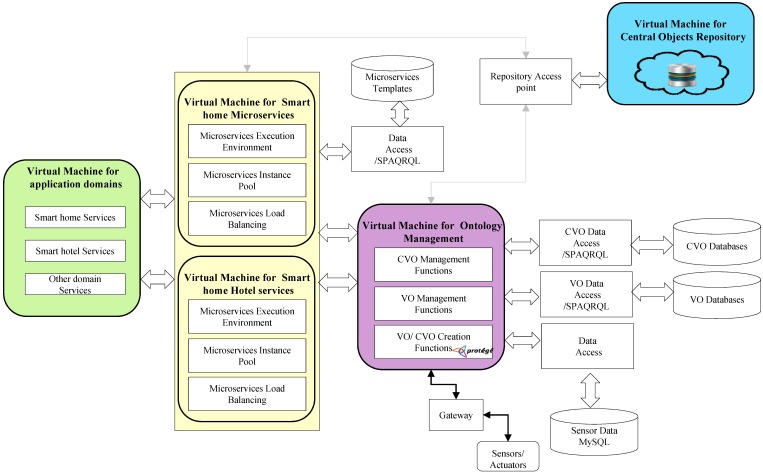
Prototype implementation model for the use case scenario.

A prototype has been implemented based on a use case scenario at advanced networking and multimedia laboratory. Figure 6 shows the microservices based prototype implementation model for a discussed use case scenario. For the microservices based prototype implementation, we installed five virtual machines (VMs) with Oracle VM virtualbox version 5.1 (https://www.virtualbox.org/wiki/Downloads) on the Ubuntu Server (https://www.ubuntu.com/server) operating system. The first VM contains domain specific application services (i.e., in our case, smart home domain services, and smart hotel domain services). The second and third VMs contain microservices and microservices related functionalities for smart home and smart hotel application domains. In the second and third VMs, microservices execution environment, microservices instance pool, and microservices load balancing functions are deployed. Microservices template databases for smart home and smart hotel application domains have also been created in these VMs. The microservices in these VMs (i.e., second and third VMs) can reuse CVOs from central objects repository, and also take relevant CVOs and VOs from ontology management VM. The fourth VM contains the virtual level management functions of WoO platform. These functions include CVO management functions, VO management functions, and VO/CVO creation functions, and this virtual machine also contains CVO databases, VO databases, and MySQL (https://www.mysql.com/) database. A triple store software is used for storing the VO and CVO ontologies and semantically annotated data. The fifth VM is used for central objects repository, which includes CVOs and VOs from both application domains. All the databases in the prototype have a separate data access point implementation. The separate data access point implementation is considered because the failure of one data access point will not fail the other ones. In the prototype implementation, microservices for both domains have also been implemented separately, because these microservices can be updated and maintained without disturbing other application domain microservices. 

In the prototype implementation, MySQL database has been used for storing the raw sensors data. The sensors, such as indoor and outdoor temperature sensors, CO_2_ sensor, humidity sensor, luminous sensor, light sensor, position sensor, accelerometer sensor, plus sensor, etc., and actuators, such as LED, HAVC, fan, etc., have been connected through the gateway. The communications among all the components and central objects repository have been achieved through RESTful Web Services. Publish/subscribe based communication with sensors actuators is achieved with RabbitMQ (https://www.rabbitmq.com/). The ontology model of VOs and CVOs has been created with protégé (https://protege.stanford.edu/download/protege/4.3/installanywhere/Web_Installers/). Apache Jena Fuseki (https://jena.apache.org/documentation/fuseki2/) SPARQL endpoint has been used for the storage of VO/CVO templates. The SPARQL query language has been used to query Apache Jena Fuseki for VO/CVO. Spring Boot (https://jaxenter.com/spring-boot-tutorial-rest-services-and-Microservice s-135148.html) has been used for the microservices development. To serve the objects and communication among the application domains, and for the deployment of RESTful web services, the Apache HTTP server (https://httpd.apache.org/download.cgi) has been used. Initially, collected data from sensors were stored in MySQL database, and, then, the data have been annotated with relevant VOs and stored in VO repository. CVOs were used to compose microservices and the Hermit 1.8.3 (http://www.hermit-reasoner.com/) reasoner has been used over the VO annotated data for further inference, relations, and knowledge.

Our prototype design and implementation have some limitations in reusing of objects with microservices. The proposed model has been applied in the use case because the smart home and smart hotel are related to each other and offer similar types of IoT services. For example, related application domains include smart hotel, smart home, smart office, etc., whereas smart industry and smart agriculture are not related to the smart home or smart hotel. However, some of the functionalities of different types of domain such as smart industry or smart agriculture can perform service creation and provisioning in the smart home or smart hotel, even with degraded quality that depends on the similarity between the requested services.

## 7. Experiments and Results

**Figure 7:**
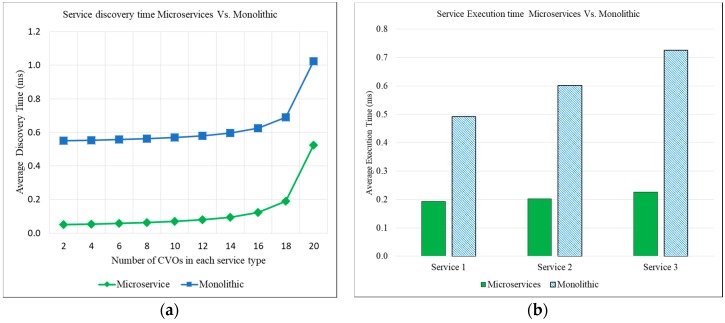
(**a**) Average discovery time for the service with the same number of CVOs with microservices vs. monolithic; and (**b**) average execution time for the service with microservices and monolithic approach.

The service implementation in monolithic approach consists a single codebase with all logic to discover, inference and execute service features with CVOs directly at the same place. The list of relevant CVOs was stored in a single service template because the logic of all service features was implemented as a single unit. In a monolithic implementation, all three services, service 1, service 2, and service 3 (Figure 7b), have a single executable binary file. The single executable binary file for all services creates easiness in the deployment of services for the IoT application domain. However, any change or update in one service affects the whole system and requires the redeployment of all services. The process of redeployment may bring down all services. The single codebase implementation and size of application may also cause the delay in service start-up time and service response time. The scalability and availability of services are two major issues of monolithic implementation, because the conflict in resource requirements and a bug in any sub function can potentially bring down the whole application domain.

In microservices approach, the implementation codes of a service were distributed to many microservices. The service accessed the required CVOs through microservices to execute service features. In this approach, the information on the required CVOs was stored in the microservices template. The different microservices were reused with different service features, due to the independency of codebase and CVOs. In our experiment, we implemented six microservices (µŞ) and reused some of the microservices in Ş_1_, Ş_2_, and Ş_3_. The first service includes four microservices, i.e., Ş_1_ = (µŞ_1_, µŞ_3_, µŞ_4,_ µŞ_6_); the second service comprises of three microservices, i.e., Ş_2_ = (µŞ_1_, µŞ_4,_ µŞ_5_); and the third service includes four microservices, i.e., Ş_3_ = (µŞ_2_, µŞ_3_, µŞ_4,_ µŞ_5_). Due to separate implementation, each service can be scaled and deployed independently. 

To measure the effectiveness of our proposed approach and its implemented prototype, the results have been verified with numerous experiments and the performance has been evaluated with respect to all system component. The system configuration for the experiment consisted of a PC with windows pro 10 64-bit, Intel Core i7-6700@3.40 GHz, 16 GB RAM, in which five VMs were installed, as discussed in the prototype detail section. During the experiments, the payload size of request and response, and execution time of microservices were considered. 

A first experiment was performed for the comparison of service discovery time with both microservices approach and monolithic approach, while keeping an equal number of CVOs in each service. The service discovery time is the time when the service request arrived and the related objects such as service, microservices and their relevant number of CVOs were searched from the relevant databases (service registry database, microservices registry database, CVO registry database, and VO registry database). In service discovery process, the IoT service request parameters were matched with the service metadata, microservice metadata, and CVO metadata.

The results of IoT service discovery time with microservices and monolithic approach are shown in Figure 7a. For comparison of service discovery time, the IoT service implemented with the microservices approach and monolithic approach. The number of CVOs in each service kept equally. Initially, in the experiment, two CVOs were used in both types of service templates and then this number was increased exponentially to 4, 6, 8, 10, 12, 14, 16, 18 and 20. During the experiment when the number of CVOs increased in the service template, the discovery time of both approaches were also increased. However, the discovery time of microservices approach increased slowly as compared with the monolithic, because of the lightweight mechanism of microservices implementation. In the experiments to support the modular and single function oriented concept of microservices, we tried to keep a minimum number of CVOs with every microservice.

The second experiment has been performed to compare the service execution time with the microservices approach and monolithic approach. The results of service execution time with both approaches are shown in Figure 7b. To compare the service execution time with microservices and monolithic approaches, three separate services were designed and implemented. Each service pair (i.e., Ş_1_ {Microservices, Monolithic}) implementation contained an equal number (2, 4, 6, 8, 10, 12, 14, 16, 18 and 20) of CVOs. The results of the experiment showed that microservices approach takes less service execution time than the monolithic because, in the microservices approach, not all objects were loaded in the cache memory, whereas, in monolithic approach, all objects are loaded into it. The other reason was lightweight container based service execution environment for every microservice. 

The results of the experiments show the importance significance in WoO platform for IoT service provisioning. Microservices is not discussed in iCore [40] and oneM2M [30] for service modularity and IoT service provisioning. The service in iCore is discussed monolithically, while our approach supports the reusable small services in terms of microservices. To increase the scalability of IoT platforms, the reusability in terms of software is discussed in Xively platform for sharing the firmware of common devices [33]. The Xively platform does not provide the reusability features at the service level. Our approach provides the reusability of objects in terms of microservices at the service level. 

## 8. Conclusions

In this paper, a novel microservices approach for enhancing the reusability of objects in WoO based IoT environment has been presented. The research design has been an interactive and agile process, in which we followed microservices pattern and semantic web technologies. A key concept in this article is the enhancement of objects’ reusability in terms of microservices and CVOs, providing the mechanism for objects sharing and semantically understanding of objects among various IoT application domains enabled with WoO based IoT environment. Microservices pattern has been followed to enhance CVO reusability and to support the rapid development and deployment. For searching and ranking of microservices, a flow chart based algorithm has been presented. For sharing the requested objects in various application domains, a central objects repository microservice, and objects repository in the cloud have been designed and implemented. For reusing the objects of other application domains through central objects repository, an algorithm for matching, mapping, and aligning has been proposed. A use case scenario of two application domains has been designed and a prototype has been implemented.

For in-depth analysis, the service discovery and service execution time with the microservices approach and monolithic approach have been compared. The results of the experiment have shown that the microservices approach takes less service discovery time and also service execution time then the monolithic in IoT service provisioning. Microservices in WoO platform support the service scalability, modularity, and objects’ reusability for intelligence IoT service provisioning. The service provisioning with microservices was not discussed in iCore, oneM2M, and Xively. The results of the proposed approach and a use case prototype implementation prove that microservices approach has great significance in IoT service provisioning and enhancement of objects’ reusability in WoO based IoT environment. Our proposed approach also has some limitations in the case of object reusing from other application domains with microservices. The proposed approach can work in related application domain, where the related IoT services are provided. However, we believe that some of the services can work partially, based on the similarity distance between the requested and responded CVOs. 

Currently, we have been working towards object reusability with microservices in various related application domains in WoO based IoT environment. In the future, we would like to extend this work to other IoT platforms.
